# An Introduction to Pathology and Morbid Anatomy

**Published:** 1889-11

**Authors:** 


**An Introduction to Pathology and Morbid Anatomy.** By T. HENRY GREEN, M. D. Sixth American, from the seventh English, edition. Revised and Enlarged by STANLEY BOYD, M. B., B. S., F. R. C. S., London, England. Philadelphia: Lea Brothers & Co. Pp. 539. 1889.

As a popular text-book on Pathology, perhaps none can excel Green's, as the number of editions through which this book has passed easily testifies. The seventh edition, edited under the direction of Dr. Boyd, has been in many places rewritten, in order to keep pace with recent pathological and bacteriological observations. The book has otherwise been improved by the addition of new illustrations and the use of larger type.

In the introduction, the writer dwells upon the action of cells in a healthy state, and their transformation into disease. The question as to whether the nervous system influences those chemical changes in which the life of cells, other than gland, muscle, and nerve, consists, remains unanswered; the author inclines to the belief that it most probably does.

Disease, or the abnormal performance of function by one or more organs or tissues, is discussed from its different standpoints, before proceeding with the special forms of disease. The opening chapters treat of Necrosis, or nutrition arrested; Degeneration, or nutrition impaired; and Hypertrophy, or nutrition increased.

The Neoplasms are next taken up, and, in a clear and masterly style, are made interesting reading for those who have occasion to refer to these subjects. Not only through the style of the writer, but in typographical arrangement and selection of illustrations does this treatise owe much of its popularity.

The chapters on Fever and Inflammation embrace the latest theories in regard to these processes; the writer seems inclined to accept the views of MacAlister, who considers thermogenesis as the result of tissue oxidation under control of the cerebro-spinal system of nerves.

Under the Infective Granulomata are discussed Tubercles and Tuberculosis, Syphilis, Scrofula, and Leprosy.

Inflammation of the Special Tissues and Organs comprises the several inflammatory conditions, and although treated rather briefly, yet are sufficient to convey to the reader the most salient points of their pathological character.

The closing chapters deal with the Vegetable Parasites, their natural history, mode of life, directions for their detection and cultivation.

As a text-book, this pathology is to be recommended. Without going into the finer detail of pathological changes, it nevertheless presents the subject as complete and concise as is consistent with a work which claims to be nothing more than an introduction to the study of pathology.

The publishers have left nothing undone to make this work, from a typographical standpoint, as attractive as possible, and is a credit to the house. W. C. K.

